# Threefoldness

**DOI:** 10.1007/s11098-017-0860-2

**Published:** 2017-01-23

**Authors:** Bence Nanay

**Affiliations:** 1 Centre for Philosophical Psychology, University of Antwerp, D 413, Grote Kauwenberg 18, 2000 Antwerp, Belgium; 2 Peterhouse, University of Cambridge, Cambridge, CB2 1RD UK

**Keywords:** Picture perception, Picture surface, Depiction, Twofoldness, Attention

## Abstract

Theories of picture perception aim to understand our perceptual relation to both the picture surface and the depicted object. I argue that we should talk about not two, but three entities when understanding picture perception: (A) the picture surface, (B) the three dimensional object the picture surface visually encodes and (C) the three dimensional depicted object. As (B) and (C) can come apart, we get a more complex picture of picture perception than normally assumed and one where the notion of twofoldness, which has played an important albeit controversial role in understanding picture perception is replaced by the concept of threefoldness.

## Introduction

How does my mind work if I see an apple in a picture? And how is this mental state different from my mental state when I see an apple face to face?

It seems that when we are looking at a picture we see not one but two things: the depicted apple and the picture of the apple.^1^ The two dimensional picture surface (which is the actual object in front of you) and the three dimensional object depicted in the picture. So one crucial question any account of picture perception needs to clarify is whether we really do see both of these things and if so, how it is possible to see two things at the same time (at the same region of my visual field).

There seem to be only three options here:

i. We only see the picture surface, not the depicted object
ii. We only see the depicted object, not the picture surface
iii. We see both the picture surface and the depicted object

Option (iii) itself comes in two very different forms:

- We see both the picture surface and the depicted object but we alternate between seeing the surface and seeing the depicted object
- We see both the picture surface and the depicted object and we see them simultaneously

According to option (i), we do not really see the depicted object. As it is not present, maybe we only imagine that it is there. Or maybe we imagine our experience of the picture surface to be an experience of the depicted object (this is Walton 1990’s account). But we do not strictly speaking see the depicted object. There are many challenges to making it precise what this ‘imagining one’s experience of the surface to be of the depicted object’ means. Moreover, it is not at all clear whether this extremely complex imaginative episode is something we humans are capable of at all, let alone all those non-human animals that are apparently capable of picture perception. Further, if we go down this route, we can no longer say that seeing an apple in a picture is one way of seeing an apple (or, having a perceptual experience of the apple as seen in the picture is one way of having a perceptual experience of the apple). Instead, it is a way of imagining seeing the apple.

Option (ii) denies that we really see the picture surface. This is an odd and somewhat desperate view, as the picture surface is right in front of us and we are staring at it. There is an important example of seeing pictures where the surface does not seem to figure in our perceptual experience, and that is the way we are meant to perceive *trompe l’oeil* pictures—at least for a split second. *Trompe l’oeil* paintings deceive the eye (hence the name): they fool us into thinking that we see the depicted object face to face—before realizing that we see a picture. But not all pictures are *trompe l’oeil* pictures. So even if it is true that we only see the depicted object but not the surface when looking at (and being fooled by) *trompe l’oeil* pictures, this is clearly not true in general.

Option (iii (a)) is normally attributed (rightly or wrongly) to Ernst Gombrich. His account of picture perception is that we see both the surface and the depicted object, but we never see the two at the same time. We oscillate between seeing the canvas and seeing the depicted scene. While I described this view as a case where we see both the picture surface and the depicted scene, it may be more appropriate to describe it as a way of combining (i) and (ii). Specifically, the proposal is that our perceptual state oscillates between (i) and (ii). But then this view will inherit at least some of the problems of option (i) and (ii).

Finally, the most widely discussed way of thinking about picture perception is (iii (b)): we simultaneously see both the two dimensional picture surface and the three dimensional depicted scene. Option (iii (b)) is often labelled as the Twofoldness Claim. When we see something in a picture we have a twofold perceptual state: we see the surface and the depicted object simultaneously (see Wollheim 1980; Walton 1990, pp. 300–301; Walton 2002, p. 33; Nanay 2005; Feagin 1998; Levinson 1998; see also Hopkins 1998, esp. pp. 15–17; Maynard 1994, esp. pp. 158–159; see also Lopes 2005; chapter 1; Kulvicki 2006, pp. 172–173 for somewhat critical overviews).

## The Twofoldness Claim

Again, the Twofoldness Claim, that is, option (iii (b)) is that when we see something in a picture we have a twofold perceptual state: we see the surface and the depicted object simultaneously. There is something odd about the Twofoldness Claim as it stands: if we see these two very different things simultaneously, how is it possible that our visual experience is not disjointed (or confused)?

Robert Hopkins argues against the Twofoldness Claim by pointing out that it does not capture the phenomenology of picture perception (Hopkins 2012). If it were the case that we have perceptual experience of two very different things simultaneously (the two dimensional surface in our egocentric space and the depicted scene not in our egocentric space), this would lead to a disjointed or confused overall perceptual experience. But this is not the kind of experience we have when we look at pictures. There may be other ways of getting around this worry, but I want to suggest that we should turn to philosophy of perception for a little help.

In outlining the four options above, I was implicitly equating ‘seeing’ with ‘having a conscious perceptual experience of’ or ‘visually attending to’. And the disjointedness worry about the Twofoldness Claim is only a worry if ‘seeing’ is interpreted this way. But we know from philosophy of perception (and from hundreds of subliminal priming experiments) that there are many ways of seeing something. First, seeing can be conscious or unconscious (Marcel 1983; Weiskrantz 1997). Second, we attend to some but not all the objects and properties we see (Mack and Rock 1998; Simmons and Chabris 1999). The relation between these two distinctions is complicated because the relation between attention and consciousness is complicated: it is not clear whether attention is necessarily conscious, for example (probably not, see Cohen et al. 2012; Jiang et al. 2006; Kentridge et al. 1999, 2008).

In order to bypass these worries, I will focus on the distinction between attending to something we see and not attending.^2^

We do not attend to most of the things that are in our visual field. In fact, we attend to very few properties of very few things most of the time. And, as the inattentional blindness experiments show, what we are attending to and what we are not attending to has an important impact on our perceptual experience (Mack and Rock 1998). Here is probably the most famous of the inattentional blindness experiments (Simmons and Chabris 1999). You see a clip where people pass a basketball around. You are supposed to count how many times the team whose members are dressed in white pass the ball among themselves. Most participants who do this fail to notice that a man in a gorilla costume walks across the screen comfortably and takes up a significant part of the screen for a long period of time. Given that their attention is directed elsewhere (to the passing of the basketball), many subjects are completely unaware of this. If there is no counting task to perform, everyone immediately notices the gorilla.

One way of interpreting this experiment is that we are not conscious of those objects or properties that we are not attending to: we are not conscious of the gorilla because we didn’t attend to it. Consciousness requires attention: if we do not attend to something, we will not become conscious of it. While I myself think that this interpretation is basically correct, I will not rely on this here—as there is an alternative interpretation according to which you were conscious of the gorilla, but you immediately forgot it. On this view, we could talk about inattentional amnesia, not inattentional blindness (see Wolfe 1999). For present purposes, all I need to assume is that the allocation of attention influences our experience of the perceived objects significantly. Crucially, priming studies show that even unattended objects (like the gorilla) can prime us (that is, it disposes us to be quicker to recognize stimuli that have something to do with gorillas Mack and Rock 1998). This shows that whether or not the unattended object is not experienced or experienced very briefly and then forgotten immediately, it is nonetheless perceived (presumably unconsciously) and that is why it can have a priming effect. In short, we can see objects with or without attending to them.

How do these considerations apply if we turn to picture perception? If we allow for different ways of seeing something, then we will have more than the four options I outlined above. We can see the picture surface with or without attending to it, and the same goes for seeing the depicted scene. A plausible interpretation of the Twofoldness Claim would be that we do not normally attend to the picture surface when seeing things in pictures. We attend to the depicted scene. Now, we *can* attend to the picture surface and this way of attending will play an important role when we try to understand the aesthetic appreciation of pictures (see Clark 1960, p. 17, pp. 26–27). But normally, we only attend to the depicted scene, not the picture surface.^3^

Remember the worry about the Twofoldness Claim that it would imply some kind of disjointed or confused experience, where properties of the depicted scene are thrown in together with the properties of the picture surface. This worry disappears if we take the picture surface to be unattended. Just as the unattended gorilla fails to show up in our experience of the basketball game, so the unattended properties of the picture surface (in normal cases) will also fail to show up in our experience of the picture. As a result, these properties are not in the position to make this experience disjointed.^4^

One may worry that while this way of thinking about picture perception manages to avoid the disjointedness objection, we only do so by endorsing another problematic assumption, again, about the phenomenology of picture perception. And this new problematic assumption is that seeing something in a picture is very similar to, maybe even indistinguishable from, seeing the same thing face to face. If the surface is not attended, whereas the depicted object is, then presumably it is the depicted object and not the surface that will show up in our phenomenology. But while this may be so with *trompe l’oeil* pictures (or maybe even with naturalistic pictures), it is clearly not the case when we are looking at impressionist, expressionist, cubist or pretty much any depictions that are not hyper-naturalistic. I think this is an important problem that all accounts of picture perception need to address, and I will do this at the end of this paper (when all the conceptual resources for doing so are at our disposal). I will argue that we can preserve the force of some of these considerations without facing some of the problems of the Twofoldness view if we add an additional third fold. But before turning twofoldness into threefoldness, I need to clarify a potential confusion about the concept of twofoldness that comes from conflating questions about picture perception and questions about the aesthetic appreciation of pictures.

## Picture perception versus the aesthetic appreciation of pictures

It is very easy to confuse the philosophical debate about picture perception with the philosophical debate about aesthetic appreciation of pictures. In fact, arguably, two of the founding fathers of the depiction literature, Ernst Gombrich and Richard Wollheim, both slide back and forth between these two very different questions.

The aesthetic appreciation of pictures is often characterized as the appreciation of pictures as pictures. Consequently, the aesthetic appreciation of pictures is clearly a subcase of picture perception. Not all instances of picture perception count as the aesthetic appreciation of the perceived picture. More often, indeed in the vast majority of cases, we see something in a picture but we do not appreciate the picture aesthetically—we do not appreciate the picture as a picture. When you are watching a sitcom or commercials on TV, when you flip through the in-flight magazine or when you look at the drawings on the emergency procedure leaflet, you see things in pictures. But you are unlikely to appreciate these pictures aesthetically (although, of course, it is not impossible). One may appreciate what is depicted in a picture without appreciating the picture as a picture.

So there are really two different questions about picture perception: what happens in our mind when we see things in pictures and what happens in our mind when we see pictures in a way that we also appreciate them aesthetically. The answer to these different questions is bound to be very different.

How is it possible then that both Gombrich and Wollheim seem to have given the same answer to these questions? Were they so confused that they failed to make this simple distinction? Or were they so highbrow that they just couldn’t look at pictures and not appreciate them aesthetically? A more natural way of reading these philosophers (and I will focus on Wollheim here) is that they had two independent proposals, one about picture perception in general and one about the aesthetic appreciation of pictures. And they—fittingly for Gombrich—oscillated between the two without noticing.

Richard Wollheim took seeing both the picture surface and the depicted object simultaneously to be a crucial feature of both picture perception in general and of the aesthetic appreciation of pictures. Assuming that he was not confusing picture perception and the aesthetic appreciation of pictures, he must have meant different things by ‘seeing’ in seeing the surface and the depicted object simultaneously when addressing the two questions. And we can and should indeed keep these two very different claims apart—as long as we use the appropriate concept of seeing.

We have seen that one way of making the proposal about simultaneous seeing work when it comes to understanding picture perception (not appreciation) is to bring in the concept of attention and to argue that while we do simultaneously see both the surface and the depicted scene, we do not simultaneously attend to both—we are only attending to the latter. But those special cases in which we are aesthetically appreciating pictures are different. Then, in addition to simultaneously seeing both the surface and the depicted scene, we also attend to the surface and the depicted scene simultaneously. Each time we see something in a picture, we see both the surface and the depicted scene. We can attend to either—although we normally attend to the latter only. But we can direct our attention to the picture surface as well as to the relation between the two. And this is what happens when we appreciate pictures aesthetically. The aesthetic appreciation of pictures is a form of picture perception where our attention is exercised in a special manner.

To make things more confusing, the account of the aesthetic appreciation of pictures I outlined in the last two paragraphs is also often labelled as the ‘Twofoldness claim’: in order to appreciate a picture aesthetically, one needs to exercise twofold attention: attending to both the picture surface and the depicted object. Richard Wollheim, who introduced the term ‘twofoldness’, as we have seen, did not make a distinction between these two claims and he did not make a distinction between the two different concepts of twofoldness that are in play when addressing these two very different questions (see Nanay 2011 on where Wollheim used which of these two concepts of twofoldness).

But then each time we talk about twofoldness, we need to make it clear which of the two concepts we have in mind. The concept of twofoldness we should take seriously in the context of picture perception is the simultaneous perceptual representation of surface and depicted object. It is twofoldness in this sense that has been argued to be necessary for picture perception. And the concept of twofoldness we should take seriously in the context of the aesthetic appreciation of pictures is the simultaneous perceptual attention devoted to both the picture surface and the depicted scene. It is twofoldness in this sense that has been argued to be very important for understanding the aesthetic appreciation of pictures.

It could be thought that it is unfortunate that both of these very different phenomena are called ‘twofoldness’, and we could blame Wollheim for confusing the reader and making two very appealing ideas much less appealing by blurring the difference between them. But I want to suggest that we may have had good reasons to run these two arguments together (acknowledging that they are different). Our perception of pictures is a twofold perceptual state: we perceive both the picture surface and the depicted object. This is true of all of our picture perception, whether or not aesthetic in nature. And this claim is silent about what we are attending to. When we see a picture, we can attend to various features of this picture. We can attend to the depicted object only: this is what happens normally. But we can also attend in a twofold manner: to both the surface and the depicted scene. If this happens, we are in the realm of the aesthetic appreciation of pictures. And for this we need twofold attention.

Allowing for an explanation of the aesthetic appreciation of pictures is an important desideratum for any account of picture perception. And the Twofoldness Claim has some impressive explanatory simplicity in this respect: the Twofoldness Claim, understood as an account of picture perception, already provides all the conceptual resources for understanding the aesthetic appreciation of pictures.

## From twofoldness to threefoldness

The Twofoldness Claim is a good starting point for understanding picture perception. I started this paper with the general question about the relation between the two things we seem to perceive when we look at a picture: the two dimensional picture surface and the three dimensional depicted object. If this is the question, then the Twofoldness Claim is an appealing answer. But I want to argue that we need to ask a different question.

When talking about picture perception, we need to consider not two, but three entities. They are the following:

- A: the two dimensional picture surface
- B: the three dimensional object the picture surface visually encodes
- C: the three dimensional depicted object

The novelty is the distinction between B and C, which many of the proponents (and critics) of the Twofoldness Claim have treated interchangeably. B and C have very different ontological status. B only exists because the picture exists: all the features of B are determined by A and A alone: by the marks on the two-dimensional surface. This is not true of C: in the case of a photograph of my grandmother, C is my grandmother and her features are not determined by A. B is a virtual object: it is fully determined by the marks on the picture surface given the rules of optics and it has only perceptible properties (whatever these may be, see Siegel 2006; Nanay 2013 for a summary).

So while B and C often do seem similar (for example in the case of naturalistic pictures), this is not always so. B and C may look very different as long as the picture is not fully naturalistic. Caricatures provide a clear example. When we look at a caricature of, say, Mick Jagger, C is Mick Jagger himself. But B, the three dimensional object the picture surface visually encodes, has very different features from Mick Jagger himself. B typically has thicker lips, for example. To use a slightly more highbrow example, in one of Henri Matisse’s portraits of his wife, Madame Matisse’s face appears to be entirely green. So B’s face is green, but C’s face (that is Madame Matisse’s face) is not green at all. A final, quite trivial example: in the case of black and white photographs, B has no color. But C does.

Again, B and C will look very different unless the picture is fully naturalistic. But even in the case of fully naturalistic pictures, B is not the same as C. B is determined by A alone, whereas C is not. B and C may look similar (in the case of naturalistic pictures), but they are different entities. Further, B does not even need to be a possible three-dimensional object. Some of Escher’s drawings encode a three-dimensional object that is a blatant impossibility. So here B would be an impossible object. C, in contrast, cannot be an impossible object.

Neither B nor C needs to be fully determinate. For example, in the case of the black and white photograph, B’s color is only minimally specified, if at all. And in the case of pictures that depict entities completely made up by the artist, B is our only guide to how C may look like.

The distinction between B and C is not entirely new. Robert Hopkins makes a somewhat similar distinction between ‘seeing-in content’ and ‘pictorial content’ (Hopkins 1998, p. 128). But note that Hopkins’s distinction has a much narrower scope—as it was introduced in order to salvage his account of depiction from potential objections (especially from the objection that the picture’s outline shape may resemble more than one depicted objects). See also Abell (2009, pp. 91–92) for a critical analysis of Hopkins’s distinction and Abell (2010, esp. pp. 83ff), where she makes a distinction between internal and external objects that is very similar to the distinction I made here (and where she also talks about the example of black and white photographs).

Lambert Wiesing, relying on Husserl, also makes a similar distinction in Wiesing (2009), where he distinguishes between image object and image subject. Wiesing, like me, makes a threefold distinction between the image-carrier, the image-object and the image-subject. These three entities would roughly correspond to what I call A, B and C: the two-dimensional picture surface, the three-dimensional object visually encoded in the picture and the depicted object.

The emphasis of Wiesing’s discussion is the image-object, which has a mere ‘artificial presence’ (Wiesing 2009, p. 35). And this emphasis also helps us to see the differences between my approach and Wiesing’s. Wiesing, again, following Husserl, takes the image-object to be the representation, which represents the image subject and he understands this representation relation as a version of resemblance (Wiesing 2009, pp. 36–38). The image carrier does not represent anything, it merely ‘displays’ the image-object and it is the image-object that does the representing. This is very different from the way pictorial representation is understood in the Wollheimian/Gombrichian tradition (one important exception is Briscoe forthcoming), where it is the picture surface that is taken to be the pictorial representation (see also Kulvicki 2014, pp. 19–20 for discussion).

Here, things get a little complicated. Assuming for the sake of simplicity that Wiesing’s threefold distinction can be mapped onto mine (more on this in Sect. 5.1 below), we can say that for Wollheim, A represents B (although given that he does not distinguish between B and C, he sometimes does seem to say that A represents C). For Wiesing, B (which is displayed by A) represents C. For me, A represents C (by means of visually encoding B). This is a major difference between my threefold distinction and Wiesing’s (another difference is discussed in Sect. 5.3 below). But the real questions in this paper are not those of pictorial representation, but of picture perception and the aesthetic appreciation of pictures. So we need to ask how we represent these three folds.

Again, we need to account for the representation of not two but three folds. The question is how they fit together. I take picture perception first and then turn to the aesthetic appreciation of pictures afterwards.

In the case of picture perception, if we want to use the insights of the twofoldness view without running into the difficulties it faces, we need to resolve the ambiguity between whether the label ‘the depicted scene’ refers to B or C: to the three dimensional object visually encoded in the surface or the actual depicted scene.

And I want to resolve this ambiguity in favour of B. It is B, the three dimensional object visually encoded in the surface, that we perceive. And we also perceive A, the picture surface. This is, so far, not a threefold, but a twofold view of picture perception. C does not have to be perceived and sometimes it may not even be represented either. If I don’t know how Mick Jagger looks, I will still perceive a person in the picture when I’m looking at the caricature. There is picture perception, but in this case, C is not even represented. But when C is represented, it is represented quasi-perceptually—by means of mental imagery.

## The three folds

More slowly, we need to go through the three folds that the Threefoldness account posits and examine how they are represented in perception (and whether they all need to be so represented). I take the three folds in turn.

### The picture surface (A)

The first fold is that of the picture surface and the threefoldness account (like the twofoldness account) claims it is perceptually represented, but not necessarily perceptually attended to.^5^

We have some empirical reasons to think that the picture surface is perceptually represented even if it is not (always) attended to. The first empirical reason is simple and straightforward (see Hagen et al. 1978), but only takes us to the claim that the picture surface is *sometimes* perceptually represented. Take two displays: an object depicted in a picture and the very same object (of the same size) behind a screen or colored glass. There is a significant difference between our judgment of the size of the object in these two displays (even if the picture depicts it in a *trompe l’oeil* manner). As the depicted/perceived object is of the same size in the two displays and, presumably (at least in the case of the *trompe l’oeil* depiction) our perception of them is also the same. But then the difference in our assessment of the object’s size must be influenced by the perception of the picture surface in the first display. This perception may be inattentive and, as a result (especially in the case of the *trompe l’oeil* picture) it may be unconscious. But unconscious perceptual states can still prime us in various ways and influence our actions, decisions and judgments.

A more complicated reason for thinking that the picture surface is perceived (but not necessarily attended to) has to do with a widely discussed topic in the psychology of picture perception: the perception of pictures from an oblique angle. An odd fact about the psychology of picture perception is that if our position changes in front of the picture, our view of the depicted object does not change (Vishwanath et al. 2005; Cutting 1987; Goldstein 1987; Halloran 1989; Pirenne 1970; Polanyi 1970; Wollheim 1980, pp. 215–216; Matthen 2005, pp. 315–317). Even if we look at a picture from an oblique angle, we don’t see the depicted scene as distorted. This is surprising and needs to be explained, as the projection of the depicted object on our retina is very different from the way it is when we look at the picture head on.

The standard way of explaining this phenomenon is to say that we are perceptually aware of the orientation of the picture surface and this awareness compensates for the oblique view: that is why we do not see the depicted object as distorted. This proposal goes back at least as far as Pirenne 1970’s analysis, allegedly inspired by a letter by Albert Einstein (see Pirenne 1970, pp. 99f).

I simplified this problem significantly (see Kulvicki 2006; Busey et al. 1990; Maynard 1996; Nanay 2011 for less simplified versions; see also Koenderink et al. 2004, p. 526 for a dissenting view). There are cases where there is no such compensation: when we are looking at ceiling frescos from an oblique angle, for example, we do see the depicted scene as distorted. And this difference may give us a clue about *how* the picture surface is represented in perception. But even bracketing these complications, we can conclude that the picture surface is perceptually represented and that is the reason why perceiving pictures from an oblique angle does not lead to distortions. This was one of Wollheim’s original reasons for talking about the simultaneous perception of surface and the depicted object (Wollheim 1980, pp. 215–216).

### The three-dimensional object visually encoded in the surface (B)

It may seem uncontroversial that the three-dimensional object visually encoded in the surface (B) is also perceived. When I see an apple in a picture, an apple shows up in my experience somehow. But it is not clear whether it is B (three-dimensional object visually encoded in the surface) or C (the actual depicted apple) that shows up in my experience. I will come back to this question at the end of the subsection—until then, I want to remain noncommittal about this and will just say ‘apple’ as a placeholder for ‘B or C’.

The real question is whether the apple shows up in my *perceptual* experience—and this is far from being clear. In fact, those who insist that imagination plays a role in perceiving pictures will deny this. They will say that we do not perceptually experience the apple: we only experience the surface and we imagine our experience of the surface to be the experience of the apple. But the experience of the apple is an imagined experience—not a perceptual one (Walton 1990).

Further, it may also be questioned whether we in fact need to experience the apple—perceptually or non-perceptually. Perception, as we have seen, can be conscious or unconscious. And picture perception can also be conscious or unconscious. Many (even most) of the experiments that demonstrate unconscious perceptual processes (for example, in unilateral neglect patients and blindsight patients as well as in the subliminal priming or inattentional blindness paradigm) are in fact conducted on subjects facing pictures (Strahan et al. 2002; Eimer and Schlaghecken 2003; Greenwald et al. 1996). Thus, any general account of picture perception, psychological or philosophical, should be applicable to both the conscious and the unconscious instances of picture perception. In the case of unconscious picture perception, we do not experience anything—we do not experience the apple either. But we do perceive the apple unconsciously: it is the perception of the apple (and not of the marks on the surface) that primes us to behave in certain ways without knowing that we have encountered the apple.

And this emphasis on unconscious picture perception may give us a reason to mistrust the imagination-based accounts of picture perception. If picture perception is taken to be conscious, then, in spite of all the criticisms of imagination-based accounts, we can at least make sense of the idea of imagining one experience to be another: of imagining the experience of the picture surface to be the experience of the apple. But it is difficult to even formulate this account in the case of unconscious picture perception. Even if we allow for the possibility that mental imagery can be unconscious (Nanay 2010b; Philips 2014), imagining one experience to be another is an imaginative episode that seems by definition conscious (as what we imagine to be something else is something conscious: an experience). The imagination-based accounts of picture perception may or may not work for conscious picture perception; but they are extremely unlikely to work for unconscious picture perception.

But dismissing the imagination-based accounts of picture perception will not give us any positive reason to think that the apple is *perceptually* experienced (in the case of conscious picture perception). It is difficult to tell apart perceptual experiences from non-perceptual ones. But regardless of how strict one is about the perceptual versus non-perceptual phenomenology distinction, we have good reason to hold that the apple is part of our perceptual phenomenology—it is perceptually experienced.

Consider so-called ‘aspect dawning’ pictures (Lopes 2005), like the famous picture of the Dalmatian. When you look at this picture, first all you see is a bunch of black patches in front of a white background. But eventually you see a Dalmatian in this picture. Where a moment ago all you saw were patches now suddenly you see a dog in the picture. Further, all the contours of the dog that you now see are illusory contours—like the sides of the Kanizsa triangle: there are no marks on the paper that would correspond to the contours of the Dalmatian (but see Cavedon-Taylor 2011 for some important differences between these two different kinds of illusory contours). In other words, what makes pictures of this kind special is that before you get to see the dog, you do not see these illusory contours—you see them only once you see the dog in the picture. Your phenomenology clearly changes when you suddenly get to see the dog.

But the question is whether your *perceptual* phenomenology changes. Suppose it doesn’t. In this case, your perceptual phenomenology would have to be what it was when you thought you were looking at the nonfigurative marks on the paper. All the changes in your phenomenology after you recognized that this is a picture of a dog and not an abstract composition are changes in your non-perceptual phenomenology. Even if we assume that the dog itself is not perceptually experienced, the surface properties are clearly very differently experienced after the transition—the illusory contours, for example, are only experienced afterwards. And once one experiences the dog, it is not possible not to be aware of these illusory contours. Thus, even if we restrict the perceptual phenomenology to the picture surface and exclude the dog, the perceptual phenomenology still changes as a result of seeing something in the picture.^6^ But then the initial assumption, namely, that the dog is part of our non-perceptual phenomenology, becomes completely ad hoc: we would need to postulate all the following processes in order to hold onto this assumption: we perceptually experience the picture surface, we non-perceptually experience the dog and this non-perceptual experience then modifies our perceptual experience of the picture surface (providing the illusory contours, for example). It may not be impossible to argue for this way of describing the case, but it entails the ad hoc postulation of a non-perceptual experience, and a top-down influence from this experience to the perceptual experience. A non-ad hoc way of describing how aspect dawning pictures work would be to say that the dog is part of our perceptual phenomenology and it is the perceptual experience of the Dalmatian that makes it possible for us to perceptually experience the illusory contours (that we did not experience before we became aware of the dog). No need to postulate either an ad hoc non-perceptual experience or an ad hoc top-down influence.

Does this argument show that B is perceived or that C is perceived? It should be clear that if this argument goes through, it only shows that B is perceived. It is possible that while B is perceived, C is not—it is non-perceptually represented—see below. The argument I gave in this subsection is consistent with this view. If this argument shows that the ‘dog’ or the ‘apple’ is perceived, this is to be understood as claims about B: about the three dimensional object visually encoded in the surface: the argument shows that we perceive the three dimensional object visually encoded in the surface.

### The depicted object (C)

So far I argued that A (the picture surface) and B (the three dimensional object visually encoded in the surface) are perceptually represented when we see things in pictures. However, the treatment of third fold, C, is more complicated.

First of all, C does not have to be perceived and sometimes it may not even be represented either. If I don’t know how Mick Jagger looks, I will still perceive a person in the picture when I’m looking at the caricature. There is picture perception, but in this case, C is not even represented.

When I do recognize the picture as the caricature of Mick Jagger, then C is represented, but it is presumably not perceptually represented: I am not perceiving Mick Jagger himself. But then how can we explain that when I recognize the picture as the caricature of Mick Jagger, my phenomenology changes?

I didn’t represent Mick Jagger before. I represent him now. And this changes my phenomenology. The question is how Mick Jagger is represented. I will argue that Mick Jagger (and C in general) is quasi-perceptually represented: represented by mental imagery (see Nanay forthcoming on mental imagery).

The first thing I need to argue for is that C is not represented non-perceptually. A straightforward alternative to my view would be to say that C merely shows up in our judgment—neither perceptually nor quasi-perceptually. This is Edmund Husserl’s and Lambert Wiesing’s view (see Wiesing 2009, pp. 70–78).^7^ (And this constitutes another major difference between Wiesing’s threefold distinction and mine.)

An initial problem with this line of the argument is that it would also make it difficult to explain why, after having recognized Mick Jagger in the caricature, our perceptual experience of the lines and shapes of the surface (i.e., of A) changes. The proponent of this view could posit a non-perceptual experience with cognitive phenomenology, which then has some kind of top-down effect on our perceptual phenomenology, but this would lead this view into more and more complicated and more and more ad hoc ways of describing what is going on in situations of this kind (further complicated in the case of Brewer’s claim by findings about how patterns of eye movements and Gestalt switches are correlated see Einhäuser et al. 2004).

Further, and more importantly, this way of thinking about C contradicts some empirical findings. As we have seen, when we see black and white photographs, B is a three dimensional object visually encoded in the picture that has no color and C is the colorful depicted object. So recognizing that what is depicted in a black and white photograph is not grey but, say, red or yellow is an instance of recognizing C. And this can be and has been empirically studied—for more than 50 years. Here is a famous experiment (Delk and Fillenbaum 1965; see also Hansen et al. 2006; Witzel et al. 2011 for more recent and methodologically more rigorous studies): if we have to match the color of a picture of an orange heart to color samples, we match it differently (closer to the red end of the spectrum) from the way we match the color of a picture of some other, orange shapes. This shows that our recognition of the object in question (the heart) influences the color we experience it as having. So when we recognize C, we perceive the color of the surface to be different from before (when we haven’t recognized C). But given that color is one of the few properties that is widely agreed to be perceptually represented, this means that representing C can and does change our perceptual experience.

This doesn’t in itself show that C is represented quasi-perceptually as it would be possible that the non-perceptual representation of C influences, in a top-down manner, our perceptual state. The problem here, again, is twofold: the blatant ad hoc nature of this proposal and its conflict with empirical findings. In an experimental setup that is similar to the one in the Delk and Fillenbaum study, subjects were put in the fMRI scanner and the activation in the visual cortex, including the primary visual cortex was different in those cases where the subject recognized C and in the cases where she didn’t (Bannert and Bartels 2013). This means that those who insist that C is represented non-perceptually (by a judgement), would need to posit a top-down influence from some non-perceptual states to the primary visual cortex. And while the primary visual cortex is subject to various attentional and crossmodal influences, it is highly implausible that it would get direct input from our judgments.

How can we explain the change in the perceived color (and in cortical activity) in this example then? How does the representation of C influence the perceived color (and the cortical activity)? A straightforward proposal would be to say that it is the mental imagery of C that influences the perceived color. You have a (not necessarily very salient) mental imagery of the heart and this mental imagery (and the color red that shows up in it) influences your perceptual experience of the orange heart-shape (that is, it influences your perceptual phenomenology). Similarly, when you recognize Mick Jagger, you have a (not necessarily very salient) mental imagery of Mick Jagger and this imagery influences the way you see the caricature.^8^

This view was explicitly defended in Macpherson (2012) as an indirect mechanism for cognitive penetration: mental imagery mediates cognitive penetration (in these Delk and Fillenbaum cases). But one doesn’t need to take sides in the Byzantine cognitive penetrability debate to hold that mental imagery influences our perceptual experience. And if C is represented by mental imagery, this can explain why it changes cortical processing of, say, color, as mental imagery is widely held to influence cortical processing, including processing in the primary visual cortex (see Kosslyn et al. 2006 for a summary).

So this gives us the following picture: we have two perceptual states and (at least in some instances of picture perception) also a quasi-perceptual state: the perceptual representation of A and the perceptual representation of B, and we also have the quasi-perceptual representation (that is the mental imagery) of C.^9^ And in order to explain the phenomenology of seeing this picture as a caricature of Mick Jagger, we need to take all three of these perceptual/quasi-perceptual states into consideration.

What makes this view of picture perception a threefoldness account (and not a twofoldness account)? This view claims that there are two perceptual states only that are involved in picture perception, not three: the perceptual representations of A and of B. And some of the time (when C is not represented at all), this is the end of the story: two folds only. But some other times, when C is represented, we need to talk about not two but three perceptual/quasi-perceptual states: the perceptual representations of A and B and the mental imagery of C. The move of bringing in three and not two folds is important for three reasons. First, it allows us to identify the perceptually represented folds as A and B (and not as A and C) and second, it explains the phenomenological difference between not recognizing C in the picture and recognizing it—in order to do so, we need to postulate a quasi-perceptual representation of C. But, again, this quasi-perceptual representation of C is not a necessary feature of picture perception.

The third reason why this is a threefoldness account becomes clear if we now turn from the question of picture perception to the question of the aesthetic appreciation of pictures. The first thing to note is that which one of these perceptual/quasi-perceptual states (that are all part of our overall mental state when perceiving pictures) is the most salient (and which ones remain unconscious) depends on our pictorial interests. Correspondingly, we can attend to any of A, B and C and any of the relations between them: we can attend to C, for example, if we want to find out how Mick Jagger or Madame Matisse looks. We can attend to the relation between B and C, when, for example, we want to assess how good the caricature is (or how naturalistic a picture is). And we can attend to the relation between A and B if we are interested in the way the marks on the surface give rise to three-dimensional features. Attending to the relation between A and B has received a lot of attention lately as a crucial aspect of the aesthetic appreciation of pictures (Budd 1995, p. 58; Podro 1991, 1998; Lopes 2005; Hopkins 2010; Nanay 2010a). But attending to the relation between B and C is important for another reason: for establishing the accuracy of the picture. Further, attending to the relation between B and C can be part of the aesthetic appreciation of pictures; when appreciating pictures aesthetically, appreciating their naturalism or the lack thereof can be very important (see also Hopkins 1997 for a related argument).^10^ If it is true that one desideratum on any account of picture perception is that it should provide the conceptual resources for understanding the aesthetic appreciation of pictures, then the third fold needs to be part of any account of picture perception (because it needs to be part of any account of the aesthetic appreciation of pictures). 11

And the introduction of the third fold may also help us to understand the difference between perceiving a picture and perceiving a sculpture. In the case of perceiving figurative sculptures, we also need to consider three entities: A, B and C: the three dimensional clay/marble/bronze object (A), the three dimensional structure encoded by this (B) and the three dimensional object/person seen in the sculpture (C). While A and B can come apart (for example, in the case of bas-reliefs), they often have the same contours. So the aesthetic appreciation of sculptures often involves attending to the relation between B and C.

## Twofoldness versus threefoldness

I gave a new account of picture perception that talks about not two but three folds. One may wonder why we should multiply folds if we don’t need to. I want to conclude with two considerations about why a threefoldness account is preferable to a twofoldness account.

First, I argue that the threefoldness account is not susceptible to the objection we considered in Sect. 2 that seems to jeopardize the twofoldness claim. We are finally in the position to go back to the objection that any account of picture perception must be able to account for the phenomenal difference between seeing something in a picture and seeing the same thing face to face. If the surface is not attended, only the depicted object is, then presumably it is the depicted object and not the surface that will show up in our phenomenology—and this sounds dangerously similar to seeing the depicted object face to face. So the worry was that while the account of picture perception I outlined above may be plausible for *trompe l’oeil* pictures or maybe even with naturalistic pictures, it is clearly a crazy view when it comes to any depiction that is not hyper-naturalistic.

We can now see that this objection is based on the conflation of B and C: of the three dimensional object visually encoded by the surface and the depicted object itself. It is B, that is, the three dimensional object visually encoded by the surface, that we attend to, not C. As we have seen, the representation of C may color our perceptual experience, but C itself is not something we normally attend to. B is what we attend to and the experience of B (say, the green-faced Madame Matisse) is very different from the experience of C (that is, the pink-faced Madame Matisse), that is, the experience of the depicted person face to face.

In other words, we can maintain that when we see something in a picture, we simultaneously see both the surface and what is in the picture: we normally do so by attending to the latter and not the former. And this does not entail an experience indistinguishable from (or even similar to) the experience of seeing the depicted object face to face because what we are attending to is not the depicted object *per se*, but the depicted object as it is depicted: the three dimensional object visually encoded by the surface—B, not C.

We can, of course, also attend to the surface, as we have seen (when, for example, aesthetically appreciating pictures). And we can also attend to the depicted object itself—to C (when, for example, assessing the accuracy of the depiction). But in all cases of seeing something in the picture, we need to attend to the three dimensional object visually encoded by the surface—to B.

Finally, the second consideration in favour of the Threefoldness Claim is the following: it allows for a more nuanced picture of the aesthetic appreciation of pictures. As we have seen, one desideratum on any account of picture perception is that it should provide the conceptual resources for understanding the aesthetic appreciation of pictures. The Twofoldness Claim does this, but the resulting account of the aesthetic appreciation of pictures is not as rich as the account of the aesthetic appreciation that we would get if we talked about not two but three folds. Some properties that are very relevant for the aesthetic appreciation of pictures are, for example, relational properties between B and C. And if we accept the threefoldness view, the conceptual resources for this aspect of the aesthetic appreciation of pictures are already present in the threefoldness account of picture perception.

## Footnotes

1 One difference between seeing an apple face to face and seeing a picture of the apple is that in the former case the apple needs to be there if I am to really see it face to face (as opposed to hallucinate it). But in the case of seeing the apple in the picture, the apple does not need to be there (and, in the vast majority of cases, it is not in fact there). In this paper, I will follow the accepted terminology of glossing picture perception as seeing something in a picture. Those more precious about their use of the term ‘seeing’ may want to read it as ‘seeming to see’ (see Hopkins 2012 on this concept in the context of picture perception). I will come back to these considerations in Sect. 5.2.

2 We could also use the conscious/unconscious distinction, as long as we take the unattended stimulus to be unconscious (as we should in the light of empirical evidence, see Cohen et al. 2012; Prinz 2010; Schwitzgebel 2007 for philosophical summaries but see also Lamme 2003 for a dissenting view).

3 I myself defended at various places a specific version of this view, where the picture surface is represented dorsally (by means of the dorsal visual subsystem) and the depicted object is represented ventrally (by means of the ventral visual subsystem) (Nanay 2008, 2011, 2015). In short, the two (more or less) separate visual subsystems (see Milner and Goodale 1995; Jeannerod 1997; but see also Schenk and McIntosh 2010 for some controversies about just how separate these two subsystems are) normally represent the same properties. But in the case of picture perception they represent different properties: the dorsal visual subsystem represents the properties of the picture surface, whereas the ventral visual subsystem represents the properties of the depicted scene. What I say about picture perception in this paper is consistent with the dorsal/ventral account but it does not depend on it.

4 For a somewhat related strategy for downplaying the salience of the picture surface in our experience (in a very different conceptual framework), see Polanyi (1970).

5 This is not a trivial claim: just because the picture surface is right in front of us, this does not mean that it is perceptually represented. An alternative would be to say that we only represent the depicted scene perceptually—the picture surface is not perceived at all.

6 Further, it is not just perceptual phenomenology that changes, but even the activation of direction-specific neurons in the primary visual cortex (see Teufel et al. in press), which makes it even more problematic to consider these changes to be driven by non-perceptual mental states.

7 This also seems to be what is implied by some other way of describing the Gestalt switch in the case of seeing the duck-rabbit figure. As Bill Brewer says, for example: “[T]he difference is no change in the core subjective character of the *experience*; it rather concerns our classificatory engagement… This is [a] further phenomenology… Basic [perceptual] experiential presentation is common throughout.” (Brewer 2007, p. 93).

8 On non-salient mental imagery, see Philips (2014) and Nanay (2010b).

9 I take the concept of ‘quasi-perceptual’ to be fairly harmless: given the well-documented similarity (in terms of processing Kosslyn et al. 2006; phenomenology Perky 1910; Segal 1972) between perception and mental imagery, even if something is represented by means of mental imagery, we can say it is represented quasi-perceptually (see Lycan 1995; Matthen 2005 on the concept of quasi-perceptual states).

10 I don’t mean to suggest here that all properties that are relevant for the aesthetic appreciation of pictures are relational ones between A, B and C. Here is yet another property, also a relational one, that is also very important for our aesthetic appreciation of pictures. According to Matthew Kieran, “when we truly appreciate a work, we appreciate […] the ways in which the artistry shapes and guides our responses” (Kieran 2005, p. 213), where I take “the ways in which the artistry shapes and guides our responses” to be a relational property that is partly determined by the artistry and partly by our responses.

11 The relation between A and C may also be aesthetically relevant, for example, in the aesthetic appreciation of portraiture—where we appreciate the way the lines on the surface can capture some important feature of the depicted person (C).
